# LB19. Intramuscular therapeutic immunization targeting Rel_Mtb_/MIP-3 induces immune signatures associated with better TB control *in vivo* compared to

**DOI:** 10.1093/ofid/ofab466.1655

**Published:** 2021-12-04

**Authors:** Styliani Karanika, James Gordy, Pranita Neupane, Richard Markham, Petros Karakousis

**Affiliations:** 1 The Johns Hopkins Hospital, Baltimore, MD; 2 Johns Hopkins Bloomberg School of Public Health, Baltimore, Maryland; 3 Johns Hopkins University School of Medicine, Baltimore, Maryland; 4 Center for Tuberculosis Research, Department of Medicine, Johns Hopkins University School of Medicine, Baltimore, Maryland

## Abstract

**Background:**

Tuberculosis (TB) is one of the leading causes of death from a single infectious agent worldwide. The lengthy treatment regimen reflects the unique ability of a subpopulation of “persister” bacteria to remain in a nonreplicating state in the infected host through various adaptive strategies, including induction of the stringent response. The key stringent response enzyme, Rel_Mtb_, is essential for long-term *Mycobacterium tuberculosis* (Mtb) survival under physiologically relevant stresses *in vitro* and in animal lungs. Recently, our group has generated a therapeutic, parenteral, *rel*_*Mtb*_ DNA vaccine, which induces Rel_Mtb_-specific cellular immunity and augments the activity of the first-line drug isoniazid against active TB in mice and guinea pigs. Our group also has applied a novel vaccination strategy involving the fusion of an antigen of interest with the immature dendritic cell (iDC)-targeting chemokine MIP-3α/CCL20, which significantly enhances antigen-specific T-cell responses. We sought to determine if this iDC-targeting strategy improves the immunogenicity of the therapeutic *rel*_*Mtb*_ DNA vaccine.

**Methods:**

We cloned the *rel*_*Mtb*_ and chemokine *MIP-3α* genes into the eukaryotic expression plasmid pSectag2b. We conducted an immunogenicity study using C57BL/6J mice, comparing the T-cell responses between the *rel*_*Mtb*_*vs. MIP-3α/rel*_*Mtb*_ DNA intramuscular vaccination groups.

**Results:**

Intramuscular administration of the DNA vaccine expressing the *MIP-3α/rel*_*Mtb*_ gene fusion induced increased production of various Mtb-protective cytokines (IL-17α, IL-2, TNF-α, IFN-γ) in various mouse tissues, including the spleen, draining lymph nodes and peripheral blood mononuclear cells, relative to the vaccine expressing *rel*_*Mtb*_ alone.

**Conclusion:**

Intramuscular immunization with a DNA vaccine expressing *rel*_*Mtb*_/*MIP-3α* induces robust *in vivo* Mtb-protective immune signatures, suggesting this may be a promising adjunctive approach in combination with standard anti-TB therapy.

**Disclosures:**

**All Authors**: No reported disclosures

